# Trends in the distribution of socioeconomic inequalities in smoking and cessation: evidence among adults aged 18 ~ 59 from China Family Panel Studies data

**DOI:** 10.1186/s12939-023-01898-3

**Published:** 2023-05-11

**Authors:** Ming Zhao Huang, Tai Yi Liu, Zhong Min Zhang, Fujian Song, Ting Chen

**Affiliations:** 1 Institute of Social Development and Health Management, Hubei Province Key Laboratory of Occupational Hazard Identification and Control, School of Public Health, Wuhan University of Science and Technology, Wuhan, 430065 China; 2 Suzhou Center for Disease Control and Prevention, Suzhou, China; 3 Norwich Medical School, University of East Anglia, Norwich, Norfolk, UK

**Keywords:** Smoking, Tobacco control, Socioeconomic status, Inequality, Adult, China

## Abstract

**Introduction:**

Cigarette smoking is usually more prevalent among those with a lower socioeconomic status (SES), which can be driven by inequalities in the initiation and cessation of smoking, giving rise to SES disparities in health. This study aimed to gauge the SES inequalities in smoking related behaviours and their evolving trends based on a nationally representative database.

**Method:**

Data were extracted from repeated cross-sectional China Family Panel Studies (CFPS) of adults aged ≥18 and <60 years in 2012, 2014, 2016 and 2018. SES was constructed by principal component analysis based on income, education and occupation. Regression-based odds ratios and coefficients as the relative effect index of inequality were applied to quantify the degree of socioeconomic inequality in smoking related behaviours and to adjust for possible confounding factors. Multivariable regressions were utilized to explore the temporal trends in smoking inequalities.

**Results:**

The smoking prevalence among men decreased from 61.16% to 2012 to 57.88% in 2018, cigarette consumption among current smokers declined from 16.71 to 15.49 cigs/per day, and the cessation rate increased from 17.55% to 24.08%. Cigarette consumption for women decreased from 13.39 in 2012 to 11.01 cigs/per day in 2018. Smoking prevalence showed significant SES inequalities among men and women from 2012 to 2018 (men: *OR*_*20*12_ (95%CI)= 0.72 (0.63, 0.83), *OR*_*20*14_ = 0.60 (0.52, 0.69), *OR*_*2016*_ = 0.58 (0.50, 0.67), *OR*_*2018*_ = 0.56 (0.48, 0.66); women: *OR*_*2012*_ = 0.63 (0.41, 0.97), *OR*_*2014*_ = 0.50 (0.32, 0.79), *OR*_*2016*_ = 0.44 (0.26,  0.73), *OR*_*2018*_ = 0.50 (0.30,  0.85)). Cigarette consumption showed significant SES inequalities among men from 2012 to 2018 (*β*_*2012*_*=*-1.39 (-2.22, -0.57), *β*_*2014*_*=*-2.37 (-3.23, -1.50), *β*_*2016*_*=*-2.35 (-3.25, -1.44), *β*_*2018*_*=*-2.91 (-3.86, -1.97)). In 2018, inequality emerged in smoking cessation rates among men and smoking intensity among women. However, all tests for trends in changes over time were not statistically significant (*P* varied from 0.072 to 0.602).

**Conclusion:**

The smoking prevalence declined between 2012 and 2018 in China. However, SES inequalities in smoking persist, while socioeconomic inequalities in smoking were not alleviated among adults aged 18 ~ 59 in China. Tobacco control measures should be implemented by giving more attention to people with lower SES who are more vulnerable to tobacco use.

**Supplementary Information:**

The online version contains supplementary material available at 10.1186/s12939-023-01898-3.

## Background

Cigarette smoking is a major contributor to numerous preventable diseases [1]. By 2030, tobacco use is projected to cause 8.3 million deaths, accounting for 10% of all-cause mortality worldwide [2]. There are more than 300 million smokers in China, accounting for one-third of the world’s smokers [3]. The annual mortality toll from smoking-related diseases is thought to exceed one million in China, [4] and the direct economic burden of smoking increased from 42.8 billion RMB in 2008 (US$6.16 billion, 1 US$=6.95 RMB) to 82.63 billion RMB in 2018 (US$12.48 billion, 1 US$=6.62 RMB ), with an average annual growth rate of 6.8% [5]. Considering the enormous number of smokers and the immense health risk attributable to smoking, tobacco control and smoking cessation campaigns have been launched, including advertisement bans, smoke-free laws and tax increases. However, evidence in high-income countries (HICs) shows that some tobacco control interventions (e.g., health education, smoke-free laws, mass media campaigns) are more effective for people with higher socioeconomic status (SES) and worsened socioeconomic inequalities in tobacco use, [6, 7] although the overall smoking rate has declined. This increased inequality may lead to higher disease, disability and premature death incidences for lower SES groups.

People with a lower SES are more susceptible to the effects of tobacco and usually spend a higher fraction of their income on tobacco and the treatment of tobacco-related diseases [8]. In addition, poor health due to tobacco use can result in reduced economic productivity, household bankruptcy, and impoverished families, which is another major economic burden on low SES tobacco users [9]. Nevertheless, lower SES is associated with greater use of cigarettes and other tobacco products as well as lower odds of quitting tobacco use, with disparities increasing over time [10]. Therefore, this socioeconomic inequality in smoking may lead to more economic and health burdens among those with lower SES, which may further worsen health and socioeconomic inequalities.

This SES pattern in tobacco use, jointly with its health and economic consequences, is mounting evidence in the context of low- and middle-income countries (LMICs) and HICs, although tobacco use and the burden of tobacco-related diseases in many countries have greatly decreased [11]. Many studies on SES inequalities in smoking have been conducted in HICs and in some low- and lower-middle-income countries, while there have been limited studies in upper-middle-income countries where smoking prevalence is often the highest [12]. Several studies have explored the association between SES and smoking, [13] but few studies have assessed the trends in SES inequalities in smoking in China. LIU TY et al. (2023) examined the smoking inequality in 2011 and 2018, and Ya F et al. (2018) explored the SES inequality and its potential risk factors in cigarette consumptions using concentration index and its decomposition methods in 2013. However, these researches focused on adults aged ≥ 45 years, while did not take into account individuals aged <45 years, nor consider the inequality of smoking cessation [14, 15]. Available data have consistently demonstrated a decrease in tobacco use with comprehensive and effective tobacco control measures, [4] while further exploration is needed in the research of smoking inequality and trends, to gain a better understanding of the epidemiological status of smoking in China. In this study, we explored the trend in SES inequalities in current smoking and examined smoking cessation and intensity by gender in China. The distributions of smoking prevalence, cessation, and intensity across SES subgroups were evaluated to provide valuable insights into the consequences of Chinese tobacco control measures on health inequalities, and for the future development of equitable tobacco control policies.

## Method

### Data sources and sample selection

Data were drawn from the China Family Panel Studies (CFPS), a national two-year repeated cross-sectional survey tracking the sociodemographic dynamics of a representative sample covering 25 out of 31 provinces (or municipalities) and representing 95% of the total population. The CFPS was conducted by the Institute of Social Science Survey (ISSS) of Peking University, with a response rate of 84.14% for the survey and a cooperation rate of 87.01% [16]. The baseline survey was formally launched in 2010, and all family members and their potential biological or adopted children were also permanently followed. Face-to-face interviews were conducted to gather information and data at three levels—individuals, families, and communities—to reflect the changes in China’s economy, society, population, development of education, individual health, etc. Further details on the design of the CFPS, including the sampling technique, can be found elsewhere [16]. Samples were collected using a multistage, probability-proportional-to-size sampling technique. Thus, a sample weighting approach was applied to fit the Chinese sociodemographic population profile pertinent to the time each survey was conducted. After discarding invalid and missing data, the number of adults aged ≥18 and aged <60 enrolled in the CFPS was 22,872 in 2012, 21,964 in 2014, 21,413 in 2016, and 20,202 in 2018.

### Operational definition

#### Current smoking, smoking cessation and smoking intensity

The definition of smoking used by the CFPS is similar to that used in the Global Adult Tobacco Survey and the American Centers for Disease Control and Prevention [17, 18]. Current smokers were categorized as individuals who had smoked at least 100 cigarettes in their lifetime and had smoked in the past month. Smoking cessation was defined as being a former smoker who had ceased smoking at the time of the most recent survey based on the responses to the following questions from the CFPS questionnaire: “Have you ever smoked in the past month?” and “When did you quit smoking?”. Smoking intensity was defined as the average daily cigarette consumption in the past month by a current smoker.

#### SES measurement

Although there is no commonly acceptable method to measure SES, an accurate assessment should consider income, occupation, and education [19]. Therefore, we used individuals’ attained education level, occupation, and annual household income per capita to estimate SES. Individuals’ level of education was measured on a scale of the highest level of education attained: 0 = illiteracy, 1 = primary school, 2 = junior high school, 3 = high school, 4 = 3-year college or vocational school, and 5 = 4-year university and above. Annual household income per capita was equal to the household income divided by the family size. Occupation was measured using the Occupational Prestige Scale derived from the Chinese Standard Classification of Occupations, in which 81 occupations are rated as standardized scores from 0-100 [20]. A lower score represents the lower prestige of that occupation.

### Statistical analysis

Principal component analysis (PCA) was used to reduce a series of variables into a single SES index (the PCA method is shown in the Supplementary Materials) [21]. Based on the SES index, participants were classified into four SES quartiles. The distributions of SES scores in 2012, 2014, 2016 and 2018 based on CFPS are shown in **Figure A1**. (Supplementary Materials). To mitigate the potential impact of variations in the distribution of socioeconomic status and ensure comparability of the relative inequality indices across different years, the ‘RIIGEN’ package in Stata was used to standardize the SES indices to a common ranking scale, [22, 23] which computes a robust and comparable relative index of inequality by accounting for the proportion of the population occupying lower or higher positions in the hierarchy [24].

We studied trends in the socioeconomic inequality of smoking-related outcomes in two steps. First, socioeconomic inequality was quantified at relative scales in each survey year (2012, 2014, 2016, 2018) [24]. Multivariable logistic regressions and linear regressions were used to estimate the association of SES with smoking related outcomes (i.e., current smoking, smoking cessation and smoking intensity as dependent variables) by gender, separately. The regressions were adjusted for potential confounders, including age, residence and marital status, which were related to smoking behaviours and SES. The odds ratios and coefficients derived from the corresponding logistic and linear regression models were used as relative inequality measures. The value of odds ratio ranges from 0 to +∞, with a value < 1 indicating the behaviour is concentrated among the SES unprivileged, and the farther away from 1 represents a larger inequality. The value of coefficients ranges from -∞ to +∞, with a value < 0 indicating the average daily cigarette consumption is higher in the SES disadvantaged, and the larger the absolute value of the coefficients are, the greater the inequalities are. The confounder-adjusted prevalence and smoking intensity were estimated using marginal-effect estimation through regression models [25]. As a second step, the adjusted prevalence (i.e., predicted probabilities) of smoking-related outcomes was pooled together from four waves. We performed interaction analyses for each smoking-related outcome in which the survey year (as a continuous variable) and the interaction between the survey year and SES were additionally added into the regression models in step one. A statistically significant interaction term (survey year×SES) indicated an increase or decrease in socioeconomic inequalities in smoking-related outcomes over time [26].

All analyses in our research were weighted by using individual and cross-sectional weights adjusted for nonresponse to obtain robust and representative results. Analyses were conducted using Stata/MP V16.0 (StataCorp, TX 2016). A two-sided *P* <0.05 was considered significant.

## Results

Table A1 (Supplementary Materials) depicts the sociodemographic characteristics of adults aged 18 ~ 59 in 2012, 2014, 2016 and 2018. Table 1 summarizes the weighted prevalence of current smoking, smoking cessation and the number of daily cigarettes consumed per capita in 2012, 2014, 2016 and 2018 by gender. The prevalence of current smoking remained particularly high (> 30%) in China, although the prevalence of smoking cessation increased from 18.68% to 2012 to 25.47% in 2018, and the smoking intensity decreased from 16.59 cigs/day per capita in 2012 to 15.32 cigs/day in 2018 among smokers. Among men, the prevalence of current smoking decreased substantially from 61.16% to 2012 to 57.88% in 2016, with a significant increase in the smoking cessation rate from 17.55% to 2012 to 24.08% in 2018, and the smoking intensity decreased from 16.71 cigs/day per capita in 2012 to 15.49 cigs/day in 2018. The changes over time in smoking rates, cessation rates among women were statistically nonsignificant, while the smoking intensity decreased from 13.39 cigs/day per capita in 2012 to 11.01 cigs/day in 2018.

Table 2 presents the relative socioeconomic inequality indices for current smoking, smoking cessation and smoking intensity in 2012, 2014, 2016 and 2018. Over the 2012–2018 period, lower socioeconomic status (SES) was associated with a higher probability of being a current smoker, as indicated by the indices and 95% confidence intervals, which ranged from 0 to 1 (men: *OR*_*20*12_ = 0.72 (0.63, 0.83), *OR*_*20*14_ = 0.60 (0.52, 0.69), *OR*_*2016*_ = 0.58 (0.50, 0.67), *OR*_*2018*_ = 0.56 (0.48, 0.66); women: *OR*_*2012*_ = 0.63 (0.41, 0.97), *OR*_*2014*_ = 0.50 (0.32, 0.79), *OR*_*2016*_ = 0.44 (0.26, 0.73), *OR*_*2018*_ = 0.50 (0.30, 0.85). Similar patterns were noticed in smoking intensity for men, smokers with lower SES tended to have greater average cigarette consumption per day (*β*_*2012*_*=*-1.39 (-2.22, -0.57), *β*_*2014*_*=*-2.37 (-3.23, -1.50), *β*_*2016*_*=*-2.35 (-3.25, -1.44), *β*_*2018*_*=*-2.91 (-3.86, -1.97)). Regarding smoking cessation rates, there was no socioeconomic-related inequality in smoking cessation rates among women, as the 95% confidence intervals of relative SES inequality indices for all four years equalled 1. However, for men, statistical significance was not achieved until 2018 (*OR*_*2018*_ = 1.30 (1.05,  1.61)), suggesting that smoking cessation was unevenly distributed among individuals of higher socioeconomic status.

**Fig. 1:**
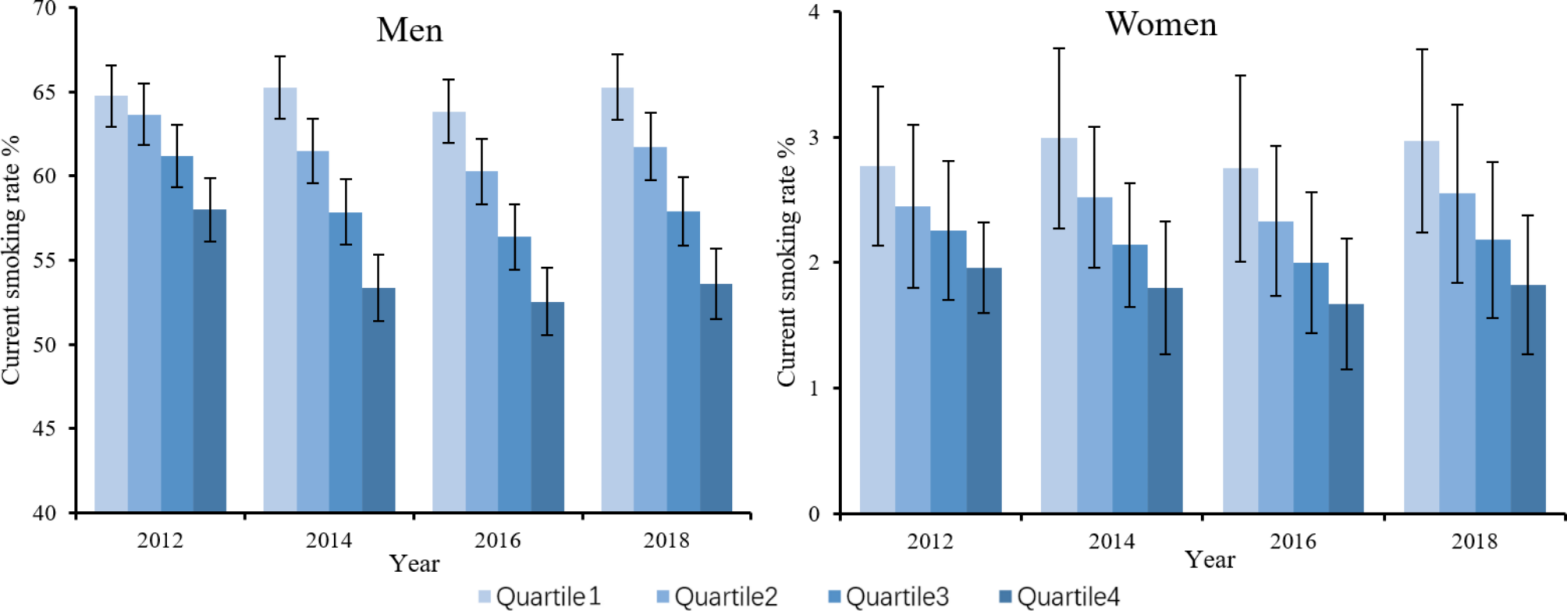
Trends in the adjusted prevalence of current smoking by socioeconomic quartile and gender. The prevalence was adjusted by SES, age, residency, and marital status; quartile 1 refers to the poorest quartile, and quartile 4 refers to the wealthiest quartile

**Fig. 2:**
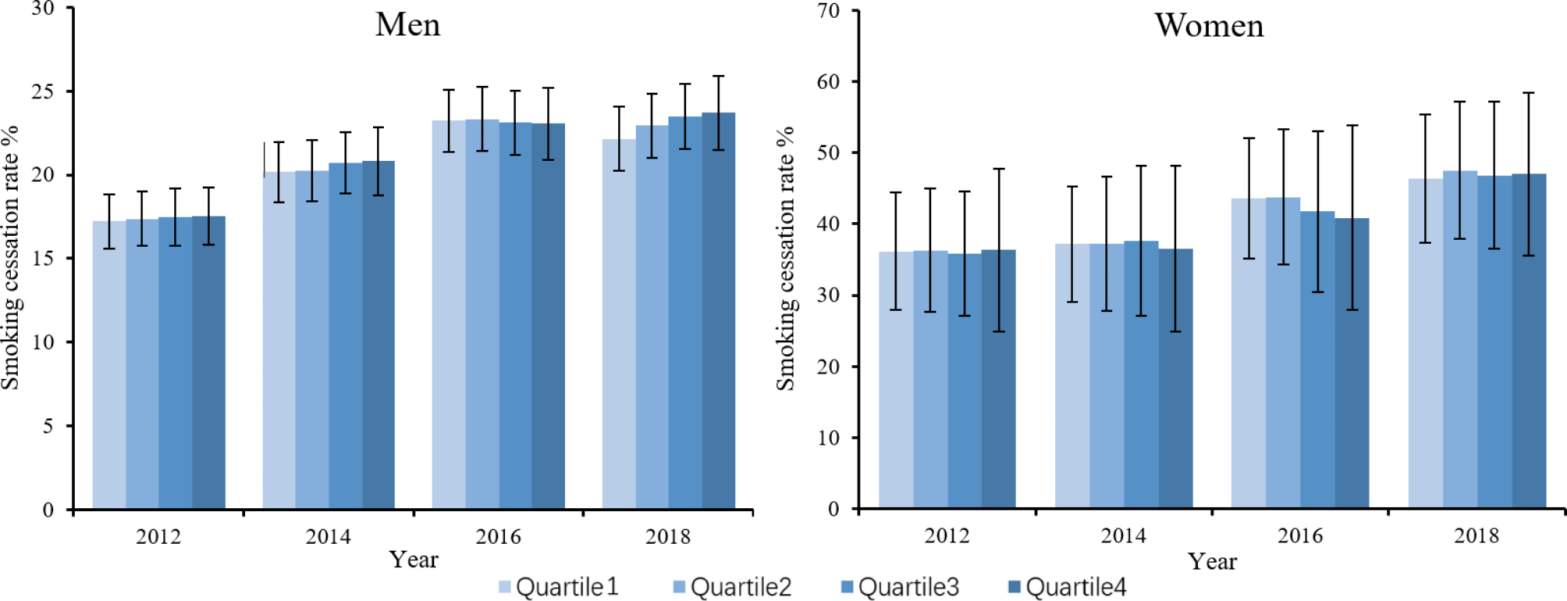
Trends in adjusted prevalence of smoking cessation by socioeconomic quartile and gender. The prevalence was adjusted by SES, age, residency, and marital status; quartile 1 refers to the poorest quartile, and quartile 4 refers to the wealthiest quartile

**Fig. 3:**
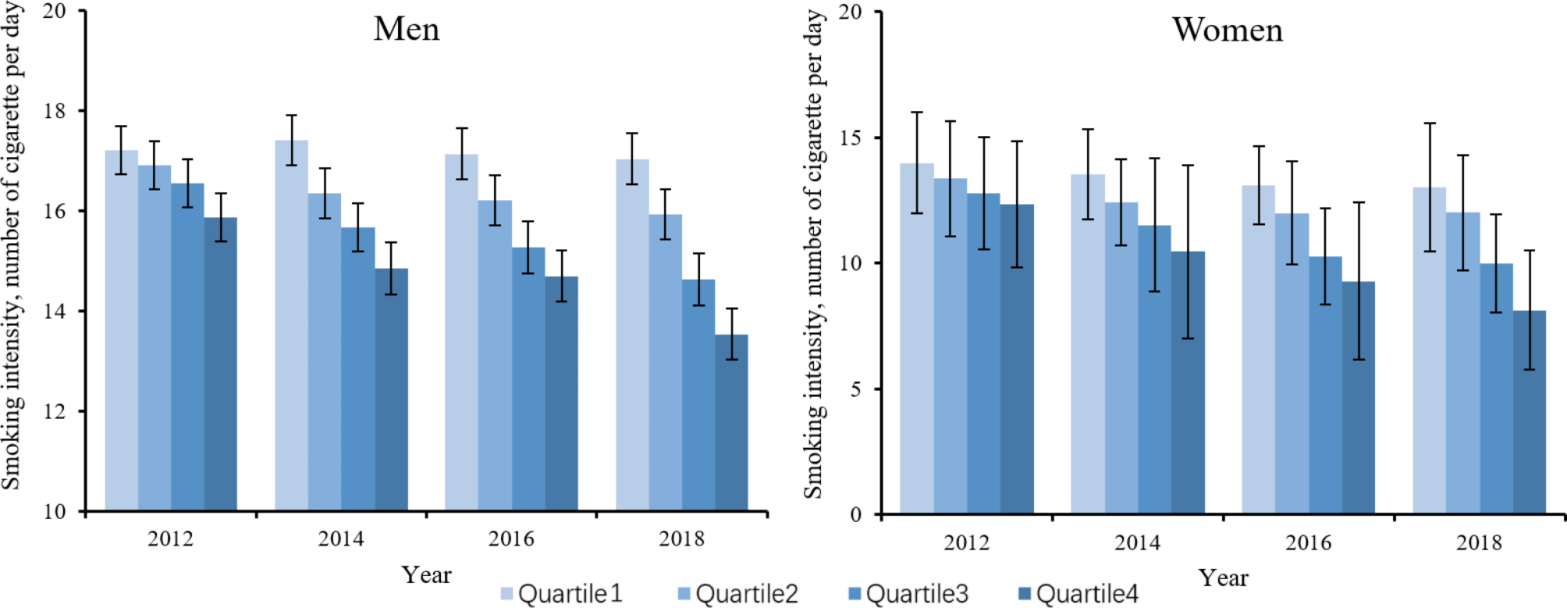
Trends in adjusted smoking intensity (cigarette consumption) by socioeconomic quartile and gender. The prevalence was adjusted by SES, age, residency, and marital status; quartile 1 refers to the poorest quartile, and quartile 4 refers to the wealthiest quartile

Figure 1 illustrates the distributions of the regression-based adjusted prevalence of current smoking by socioeconomic quartile and gender. Among men, the adjusted smoking rate was lower for the rich than for the poor. There also existed a decrease in smoking rates in the richest quartile from 2012 to 2014, 2016 and 2018, with a nonsignificant change in the poorest quartile, indicating a widening disparity between people with higher SES and those with lower SES, which was consistent with the downward trend reflected in the relative inequality indices from 2012 to 2018, while the trend over time did not reach the significance (*P* = 0.104). Figure 2 shows the adjusted distributions of the smoking cessation rates. Among men, the smoking cessation rates among different SES quartiles showed a significant increase from 2012 to 2016. However, the differences between different quartiles within each year were not statistically significant. For women, there was no significant variation in smoking cessation prevalence among different SES quartiles between different years, with a nonsignificant distribution difference within each year. Figure 3 shows the distributions of adjusted smoking intensity, which we measured with daily cigarette consumption per capita among current smokers. Among men who were currently smoking, daily cigarette consumption was higher for the poorer quartiles. There were also significant decreases in consumption in the richest 2 quartiles from 2012 to 2018, while the smoking intensity for the poorest 2 quartiles remained stable, which was consistent with the results above. Among current smokers for women, cigarette consumption was equally distributed among different SES quartiles from 2012 to 2016, whereas it was concentrated more among poorer individuals in 2018 (detailed descriptions of the smoking distribution are shown in Supplementary Materials Tables A2-4, and the crude prevalence is shown in Supplementary Materials Tables A5-7).

**Table 1:** Trends in current smoking rates, cessation rates and smoking intensity by gender from 2012 to 2018

	Year			
	2012	2014	2016	2018
**Men**				
Current smoking rate	61.16 (60.24 to 62.09)	57.34 (56.37 to 58.31)	57.15 (56.17 to 58.12)	57.88 (56.87 to 58.89)
Cessation rate	17.55 (16.71 to 18.38)	21.61 (20.36 to 22.86)	24.45 (23.48 to 25.43)	24.08 (23.08 to 25.08)
Smoking intensity	16.71 (16.47 to 16.95)	16.26 (16.01 to 16.52)	16.14 (15.88 to 16.40)	15.49 (15.24 to 15.75)
**Women**				
Current smoking rate	2.30 (2.02 to 2.58)	2.30 (2.01 to 2.59)	2.46 (2.15 to 2.77)	2.52 (2.20 to 2.85)
Cessation rate	39.44 (34.81 to 44.07)	40.28 (34.04 to 46.51)	42.08 (37.00 to 47.16)	48.39 (43.36 to 53.43)
Smoking intensity	13.39 (12.26 to 14.53)	12.30 (11.14 to 13.45)	11.26 (10.23 to 12.29)	11.01 (9.78 to 12.24)
**Total**				
Current smoking rate	30.82 (30.21 to 31.43)	29.39 (28.76 to 30.01)	29.79 (29.15 to 30.44)	31.02 (30.34 to 31.70)
Cessation rate	18.68 (17.85 to 19.51)	22.57 (21.33 to 23.80)	25.39 (24.42 to 26.36)	25.47 (24.48 to 26.46)
Smoking intensity	16.59 (16.35 to 16.82)	16.11 (15.86 to 16.35)	15.94 (15.69 to 16.19)	15.32 (15.06 to 15.57)

*The current smoking rates and cessation rates are percentages, and smoking intensity was measured by the average number of cigarettes consumed (cigs/day per capita). All of the values were adjusted by individual nonresponse and cross-sectional weights.*

**Table 2:** Relative socioeconomic inequality indices in current smoking, smoking cessation and smoking intensity by gender for adults aged 18–59 from the China Family Panel Studies 2012–2018

	Year				*P* for trend
	2012	2014	2016	2018	
**Men**					
Current smoking ^a^	**0.72 (0.63 to 0.83)**	**0.60 (0.52 to 0.69)**	**0.58 (0.50 to 0.67)**	**0.56 (0.48 to 0.66)**	0.104
Smoking cessation ^a^	1.07 (0.87 to 1.31)	1.12 (0.91 to 1.36)	1.06 (0.87 to 1.30)	**1.30 (1.05 to 1.61)**	0.285
Smoking intensity ^b^	**-1.39 (-2.22 to -0.57)**	**-2.37 (-3.23 to -1.50)**	**-2.35 (-3.25 to -1.44)**	**-2.91 (-3.86 to -1.97)**	0.072
**Women**					
Current smoking ^a^	**0.63 (0.41 to 0.97)**	**0.50 (0.32 to 0.79)**	**0.44 (0.26 to 0.73)**	**0.50 (0.30 to 0.85)**	0.602
Smoking cessation ^a^	0.77 (0.37 to 1.62)	0.98 (0.47 to 2.08)	1.15 (0.51 to 2.57)	1.68 (0.79 to 3.56)	0.216
Smoking intensity ^b^	0.51 (-3.60 to 4.62)	-0.87 (-3.16 to 4.90)	-1.01 (-4.83 to 2.80)	**-3.93 (-8.23 to -0.37)**	0.123

^*a*^ *The regression-based relative effect index was estimated according to the adjusted odds ratio of SES for smoking behaviours. Relative inequality was considered significant if the 95% confidence interval did not cross one*

^*b*^ *The regression-based relative effect index was estimated according to the adjusted coefficient of SES for smoking intensity. Relative inequality was considered significant if the 95% confidence interval did not cross zero*

*Bold indicates P < 0.05*

## Discussion

The analysis of data from the CFPS shed light on changes in smoking status between 2012 and 2018 in China. The prevalence of smoking and cessation found in this study was similar to results from the Global Adult Tobacco Survey (GATS) in China (the 2-year cessation rates were annualized by Eq. 1, Supplementary Materials Table A8), [4, 27] indicating the representativeness of the CFPS. The current smoking rate is close to 60% among men and less than 5% among women, reflecting the fact that, in East Asia, smoking is more socially acceptable for men than for women [12]. In traditional Chinese culture, smoking is perceived as a manifestation of masculinity, and offering cigarettes or smoking during social events is considered a means of demonstrating mutual respect and expressing gratitude, and tobacco companies in the Chinese market tend to emphasize advertising campaigns targeting men. Nonetheless, traditional norms dictate that smoking is inappropriate behaviour for women, as it may compromise their feminine image [28]. Our study also showed a decline in the smoking rate and an increase in the cessation rate among men, while these changes among women were not significant. One of the reasons may be the lack of antismoking campaigns that target women who currently smoke [29]. Nonetheless, the prevalence of current smoking and cessation may not reflect the whole picture of smoking status because tobacco control measures may reduce cigarette consumption rather than smoking cessation [30]. Therefore, smoking intensity as measured by the number of cigarettes smoked daily was presented by gender and SES quartile. Between 2012 and 2018, current smokers experienced a noteworthy reduction in cigarette consumption from 16.59 to 15.32 cigarettes per day. This decline may be attributed to the implementation of smoking restrictions in public spaces across some regions of China starting in 2015 [31]. Such measures have proven effective in curbing smoking intensity and reducing tobacco use prevalence [28].

Our findings show that SES inequalities in smoking persist in China. For smoking prevalence, there was a SES distribution concentrated among both poorer men and women, suggesting that people with a lower SES were more likely to smoke. Although the statistical test for this upward trend over time was not significant, these widening disparities may be worsening among men attributed to the fact that the smoking prevalence of the highest quartile decreased more than that of the other quartiles, which was in accordance with the report in some HICs [32–34]. In addition, the same pattern was observed in cigarette consumption among men who currently smoke. Men with lower SES were more likely to have a higher level of smoking intensity, while this inequality did not emerge among women smokers until 2018. Heavy smoking was more common among people with lower SES, which is similar to findings of previous studies within developed countries [35]. These results indicate that cigarette smoking has double the negative effect on socioeconomically disadvantaged individuals: they are not only more likely to smoke but also smoke more cigarettes per day [36].

Disadvantaged individuals are more vulnerable to smoking, while poor health due to smoking results in reduced SES [37]. International evidence suggests that low SES groups have the highest smoking rates because they are more likely to try smoking; for example, disadvantaged adolescents may have difficulties withstanding peer pressure and be prone to develop a regular smoking habit [38]. Moreover, some tobacco control policies are more effective for people with higher SES [36]. For instance, people with lower SES associated with a lower level of schooling are less influenced by mass media and less likely to participate in prevention programs or cessation interventions, and smoking bans are also less common in socioeconomically disadvantaged communities [39]. In addition, several studies have found an association between SES and quitting success, [40] and people with lower SES are less likely to succeed in smoking cessation [36, 39]. However, in this research, the differences in cessation rates among different SES quartiles in each year were not significant. This could be attributed to the smoking cessation indicator utilized, which is a 2-year smoking cessation rate derived from both current and past smoking statuses. This method may potentially inflate quit rates by categorizing certain individuals who have made attempts to quit as successfully having quit.

China issued its first smoke-free law in 1987, marking the beginning of antismoking. Subsequently, the Chinese National People’s Congress ratified the World Health Organization Framework Convention on Tobacco Control (WHO FCTC) on 27 August 2005, and new antismoking laws came into effect. In October 2016, an ambitious Healthy China 2030 target was announced, which included a decrease in the overall smoking rate from 27.7% to 2015 to 20% by 2030, with a series of regional tobacco control policies being promulgated. These tobacco control policies have mostly concentrated on awareness and behaviour modification initiatives, without giving attention to escalating inequalities. Several systematic reviews have documented evidence on the impact of smoking control interventions on SES inequalities in smoking [7, 36, 41, 42]. The findings of systematic reviews indicate that tobacco taxation/price measures are effective in decreasing smoking inequalities by having greater effects among people with lower SES [7]. However, China’s cigarette tax accounts for only 56% of the price of cigarettes, [43] which is much lower than the WHO recommended share of 70% of the retail price at a minimum, [44] although the tobacco excise tax was raised 4 times since the tobacco tax reform in 1994. In fact, spending on 100 cartons of cigarettes as a percentage of gross domestic product (GDP) per capita decreased from 2.0 to 1.5% in 2018 [4]. Substantially higher tobacco taxes will be needed in China to achieve the Healthy China 2030 target and curb rising inequalities [28].

Although this study provided some potentially insightful evidence that SES inequalities in cigarette smoking persist in China, there are still several limitations. First, the CFPS 2012 data indicated that the occupational prestige and educational attainment of the national population were notably lower than in subsequent years. This disparity resulted in an incomparability of socioeconomic status (SES), which may have had a partial impact on this study’s findings. In addition, data on cigarette smoking from the CFPS were self-reported and may have suffered from social desirability bias. Finally, the indicator of smoking cessation was constructed from the current and previous smoking status in different waves, which probably overestimated the smoking cessation rate and omitted some information.

## Conclusion

The overall prevalence of smoking in China has declined, especially among men. However, SES inequalities in smoking behaviour persist. People with lower socioeconomic status are more likely to be current smokers and men with lower SES tend to smoke more. More targeted tobacco control measures and policies on smoking are needed, particularly with an aim to reduce inequalities for people with lower SES.

## Electronic supplementary material

Below is the link to the electronic supplementary material.

Supplementary Material 1

## Data Availability

The data that support the findings of this study are available in the China Family Panel Studies at www.isss.pku.edu.cn/cfps/.

## Abbreviations

SES: Socioeconomic status
CFPS: China Family Panel Studies
LMIC: Low- and middle-income country
HIC: High-income country
WHO: World Health Organization
PCA: Principal component analyse

## References

[1] Jha P (2009). Avoidable global cancer deaths and total deaths from smoking. Nat Rev Cancer.

[2] Mathers CD, Loncar D (2006). Projections of global mortality and burden of disease from 2002 to 2030. PLoS Med.

[3] Wipfli H, Samet JM (2009). Global economic and health benefits of tobacco control: part 1. Clin Pharmacol Ther.

[4] CDC (2019). Report of 2018 adult smoking survey in China.

[5] Zhang YG, Wu SY (2021). [Analysis on direct economic burden of diseases attributable to smoking among chinese residents]. Chinse Journal of Hospital Statistics.

[6] Smith CE, Hill SE, Amos A (2020). Impact of population tobacco control interventions on socioeconomic inequalities in smoking: a systematic review and appraisal of future research directions. Tob Control.

[7] Brown T, Platt S, Amos A (2014). Equity impact of population-level interventions and policies to reduce smoking in adults: a systematic review. Drug Alcohol Depend.

[8] Mentis AA (2017). Social determinants of tobacco use: towards an equity lens approach. Tob Prev Cessat.

[9] Bor J, Cohen GH, Galea S (2017). Population health in an era of rising income inequality: USA, 1980–2015. Lancet.

[10] Hiscock R, Dobbie F, Bauld L. Smoking Cessation and Socioeconomic Status: An Update of Existing Evidence from a National Evaluation of English Stop Smoking Services. Biomed Res Int, 2015:274056. 10.1155/2015/274056 PMC4529910 26273602

[11] WHO (2003). WHO report on the global tobacco epidemic 2017: monitoring tobacco use and prevention policies.

[12] Mariapun J, Hairi NN, Ng CW (2019). Socioeconomic differences in Smoking and Cessation across a period of Rapid Economic Growth in an Upper-Middle-Income Country. Nicotine Tob Res.

[13] Li L, He J, Ouyang F, Qiu D, Li Y, Luo D (2021). Sociodemographic disparity in health-related behaviours and dietary habits among public workers in China: a cross-sectional study. BMJ Open.

[14] Liu T-Y, Qiu D-C, Song F, Chen T. Trends in Socio-economic inequality in Smoking among Middle-aged and older adults in China: evidence from the 2011 and 2018 China Health and Retirement Longitudinal Study. Nicotine & Tobacco Research, 2023. 10.1093/ntr/ntac158 35764073

[15] Si Y, Zhou Z, Su M, Wang X, Li D, Wang D *et al*. Socio-Economic Inequalities in Tobacco Consumption of the older adults in China: a decomposition method. Int J Environ Res Public Health. 2018, 15(7). 10.3390/ijerph15071466 PMC6069145 29997376

[16] Xie Y, Hu J (2014). An introduction to the China Family Panel Studies (CFPS). Chinese Sociological Review.

[17] Global Tobacco Surveillance System (2009). *Global adult Tobacco Survey (GATS)*: Indicator Guidelines: Definition and Syntax.

[18] Centers for Disease Control and Prevention. Adult Tobacco Use Information. 2017; https://www.cdc.gov/nchs/nhis/tobacco/tobacco_glossary.htm. Accessed September 2017.

[19] Li S, Xu Q, Xia R (2019). Relationship between SES and Academic Achievement of Junior High School students in China: the Mediating Effect of Self-Concept. Front Psychol.

[20] Li C (2005). Prestige Stratification in the Contemporary China:occupational prestige measures and socio-economic index. Sociological Research.

[21] Cao G, Cui Z, Ma Q, Wang C, Xu Y, Sun H (2020). Changes in health inequalities for patients with diabetes among middle-aged and elderly in China from 2011 to 2015. BMC Health Serv Res.

[22] Kroll LE. RIIGEN: Stata module to generate variables to compute the relative index of Inequality. Statistical Software Components, 2013.

[23] Sandoval JL, Leão T, Cullati S, Theler JM, Joost S, Humair JP (2018). Public smoking ban and socioeconomic inequalities in smoking prevalence and cessation: a cross-sectional population-based study in Geneva, Switzerland (1995–2014). Tob Control.

[24] Mackenbach JP, Kunst AE (1997). Measuring the magnitude of socio-economic inequalities in health: an overview of available measures illustrated with two examples from Europe. Soc Sci Med.

[25] Graubard BI, Korn EL (1999). Predictive margins with survey data. Biometrics.

[26] Richardson RA, Keyes KM, Medina JT, Calvo E (2020). Sociodemographic inequalities in depression among older adults: cross-sectional evidence from 18 countries. Lancet Psychiatry.

[27] Qiu D, Chen T, Liu T, Song F (2020). Smoking cessation and related factors in middle-aged and older chinese adults: evidence from a longitudinal study. PLoS One.

[28] Goodchild M, Zheng R (2019). Tobacco control and healthy China 2030. Tob Control.

[29] Morrow M, Barraclough S (2003). Tobacco control and gender in south-east Asia. Part II: Singapore and Vietnam. Health Promot Int.

[30] Lim HK, Khang YH. Tobacco price increases in Korea and their impact on socioeconomic inequalities in smoking and subsequent socioeconomic inequalities in mortality: a modelling study. Tob Control. 2020. 10.1136/tobaccocontrol-2019-055348 32220983

[31] Mackay J (2016). China: the tipping point in tobacco control. Br Med Bull.

[32] Cho HJ, Song YM, Smith GD, Ebrahim S (2004). Trends in socio-economic differentials in cigarette smoking behaviour between 1990 and 1998: a large prospective study in korean men. Public Health.

[33] Barnett R, Pearce J, Moon G (2009). Community inequality and smoking cessation in New Zealand, 1981–2006. Soc Sci Med.

[34] Daponte-Codina A, Bolívar-Muñoz J, Ocaña-Riola R, Toro-Cárdenas S, Mayoral-Cortés J (2009). Patterns of smoking according to individual social position, and to socio-economic environment in municipal areas, Spain 1987–2001. Health Place.

[35] The W (1999). Curbing the epidemic: governments and the economics of tobacco control. The World Bank. Tob Control.

[36] Hiscock R, Bauld L, Amos A, Fidler JA, Munafo M (2012). Socioeconomic status and smoking: a review. Ann N Y Acad Sci.

[37] Disease P, National Center for Chronic (2014). Health Promotion Office on S, Health: reports of the Surgeon General. The Health Consequences of Smoking—50 years of progress: a report of the Surgeon General.

[38] Petkovic J, Duench S, Trawin J, Dewidar O, Pardo Pardo J, Simeon R (2021). Behavioural interventions delivered through interactive social media for health behaviour change, health outcomes, and health equity in the adult population. Cochrane Database Syst Rev.

[39] David A, Esson K, Perucic AM, Fitzpatrick C, Blas E, Kurup AS. Tobacco use: equity and social determinants. Geneva: Switzerland. World Health Organization; 2010.

[40] Nargis N, Yong HH, Driezen P, Mbulo L, Zhao L, Fong GT (2019). Socioeconomic patterns of smoking cessation behavior in low and middle-income countries: emerging evidence from the global adult Tobacco surveys and international Tobacco control surveys. PLoS One.

[41] Thomas S, Fayter D, Misso K, Ogilvie D, Petticrew M, Sowden A (2008). Population tobacco control interventions and their effects on social inequalities in smoking: systematic review. Tob Control.

[42] Smith CE, Hill SE, Amos A. Impact of population tobacco control interventions on socioeconomic inequalities in smoking: a systematic review and appraisal of future research directions. Tob Control. 2020. 10.1136/tobaccocontrol-2020-055874 PMC8666809 32994297

[43] Du EH, Lei HC (2020). Empirical study on the impact of raising Tobacco Tax on cigarette consumption in China. Chinese Health Economics.

[44] WHO: WHO Framework Convention on Tobacco Control. Geneva, Switzerland: World Health Organization; 2003. Accessed January 21, 2015.

