# Supplementary material for: Trends in the distribution of socioeconomic inequalities in smoking and cessation: evidence among adults aged 18 ~ 59 from China Family Panel Studies data
Source: Int J Equity Health. 2023 May 11;22:86. doi: 10.1186/s12939-023-01898-3 (PMC10176762; doi:10.1186/s12939-023-01898-3)
Supplement: Supplementary file 1 — Supplementary Material 1 [file 12939_2023_1898_MOESM1_ESM.docx]

**Supplementary Materials**

**Principal Component Analysis (PCA)**

PCA is a multivariate statistical technique that is used to reduce the number of variables in a dataset into a smaller number of ‘dimensions’. In mathematical terms, from an initial set of $n$ correlated variables, PCA creates uncorrelated indices or components, where each component is a linear weighted combination of the initial variables. For example, from a set of variables $X_{1}$ through $X_{n}$,

$${PC}_{1}=a_{11}X_{1}+a_{12}X_{2}+\ldots+a_{1n}X_{n}$$

$\vdots$

$${PC}_{m}=a_{m1}X_{1}+a_{m2}X_{2}+\ldots+a_{mn}X_{n}$$

where $a_{mn}$ represents the weight for the $m$th principal component and the $n$th variable.[1]

The weights for each principal component are given by the eigenvectors of the correlation matrix, or if the original data were standardized, the covariance matrix.

The variance $(\lambda)$ for each principal component is given by the eigenvalue of the corresponding eigenvector. The components are ordered so that the first component (${PC}_{1}$) explains the largest possible amount of variation in the original data, subject to the constraint that the sum of the squared weights $(a_{11}^{2}+a_{12}^{2}+\ldots+a_{1n}^{2})$ is equal to one. As the sum of the eigenvalues equals the number of variables in the initial dataset, the proportion of the total variation in the original dataset accounted for by each principal component is given by $(\lambda_{i}/n)$. The second component (${PC}_{2}$) is completely uncorrelated with the first component and explains additional but less variation than the first component, subject to the same constraint. Subsequent components are uncorrelated with previous components; therefore, each component captures an additional dimension in the data while explaining smaller and smaller proportions of the variation in the original variables. The higher the degree of correlation among the original variables in the data, the fewer components required to capture common information.

In this study, we used participants’ level of education, occupation, and annual household income per capita to estimate SES. Participants’ level of education was measured on a scale of the highest level of education attained: 1 = illiteracy, 2 = primary school, 3 = middle school, 4 = high school, 5 = 3-year college, 6 = 4-year university and above. Annual household income per capita was equal to the household income divided by the family size. Occupation was measured using the Occupational Prestige Scale derived from the Chinese Standard Classification of Occupations, in which 81 occupations are rated as scores standardized from 0-100.[2] A lower score represents the lower prestige of that occupation.

**Figure A1** shows the SES distribution of adults aged 18~59 in China. The histogram depicts the percentage of the population (y axis) against the percentile of SES (x axis). The distribution is positively skewed, which is consistent with the distribution of China’s socioeconomic status.

**Figure A1.** The distributions of socioeconomic status among the people aged 18~59 during the CFPS in 2012, 2014, 2016 and 2018

**Table A1.** Summary statistics for adults aged 18~59 from China Family Panel Studies data

| **Variables** | **Distribution** | | | |
| --- | --- | --- | --- | --- |
|  | **2012** | **2014** | **2016** | **2018** |
| **Gender** |  |  |  |  |
| Men | 0.4811 | 0.4855 | 0.4989 | 0.4939 |
| Women | 0.5189 | 0.5145 | 0.5011 | 0.5061 |
| **Age** | 40.79 | 40.73 | 40.40 | 40.96 |
| **Residence** |  |  |  |  |
| Urban | 0.4475 | 0.4811 | 0.4945 | 0.5126 |
| Rural | 0.5525 | 0.5189 | 0.5055 | 0.4874 |
| **Marriage** |  |  |  |  |
| No | 0.1274 | 0.1352 | 0.1473 | 0.1513 |
| Yes | 0.8726 | 0.8648 | 0.8527 | 0.8487 |
| **Education Level** |  |  |  |  |
| Illiteracy | 0.2370 | 0.1682 | 0.1800 | 0.1533 |
| Primary school | 0.2141 | 0.2178 | 0.2157 | 0.1914 |
| Middle school | 0.3070 | 0.3184 | 0.3204 | 0.3290 |
| High school | 0.1502 | 0.1548 | 0.1534 | 0.1653 |
| 3-year college | 0.0572 | 0.0630 | 0.0738 | 0.0869 |
| 4-year university | 0.0345 | 0.0778 | 0.0567 | 0.0742 |
| **Household Income per Capita** | 12550.43 | 16142.61 | 23058.80 | 28715.51 |
| **Occupational Prestige** | 21.97 | 26.66 | 27.59 | 28.34 |
| ***N*** | 22,872 | 21,964 | 21,413 | 20,202 |

**Table A2.** The regression based adjusted prevalence of current smoking by SES quartile and gender.

|  | **Adjusted prevalence of current smoking and 95% confidence intervals (%)** | | | |
| --- | --- | --- | --- | --- |
|  | **2012** | **2014** | **2016** | **2018** |
| **Men** |  |  |  |  |
| Q1 | 64.75 (62.93~66.57) | 65.25 (63.37~67.13) | 63.82 (61.94~65.70) | 65.27 (63.31~67.23) |
| Q2 | 63.66 (61.83~65.49) | 61.47 (59.56~63.38) | 60.27 (58.34~62.20) | 61.75 (59.77~63.73) |
| Q3 | 61.17 (59.31~63.03) | 57.86 (55.92~59.80) | 56.39 (54.44~58.34) | 57.90 (55.89~59.91) |
| Q4 | 57.99 (56.11~59.87) | 53.38 (51.40~55.36) | 52.52(50.52~54.52) | 53.60 (51.51~55.69) |
| **Women** |  |  |  |  |
| Q1 | 2.77 (2.13~3.41) | 2.99 (2.27~3.71) | 2.75 (2.01~3.49) | 2.97 (2.24~3.70) |
| Q2 | 2.45(1.80~3.10) | 2.52 (1.96~3.08) | 2.33 (1.73~2.93) | 2.55 (1.84~3.26) |
| Q3 | 2.26 (1.71~2.81) | 2.14 (1.64~2.64) | 2.00 (1.44~2.56) | 2.18 (1.56~2.80) |
| Q4 | 1.96 (1.60~2.32) | 1.80 (1.27~2.33) | 1.67 (1.15~2.19) | 1.82 (1.27~2.37) |

**Table A3.** The regression based adjusted prevalence of 2-year smoking cessation rates by SES quartile and gender.

|  | **Adjusted prevalence of cessation rates and 95% confidence intervals (%)** | | | |
| --- | --- | --- | --- | --- |
|  | **2012** | **2014** | **2016** | **2018** |
| **Men** |  |  |  |  |
| Q1 | 17.22 (15.60~18.84) | 20.16 (18.37~21.95) | 23.23 (21.38~25.08) | 22.13 (20.21~24.05) |
| Q2 | 17.36 (15.75~18.97) | 20.24 (18.43~22.05) | 23.33 (21.39~25.27) | 22.93 (21.00~24.86) |
| Q3 | 17.46 (15.74~19.18) | 20.71 (18.89~22.53) | 23.10 (21.19~25.01) | 23.46 (21.52~25.40) |
| Q4 | 17.53 (15.84~19.22) | 20.80 (18.78~22.82) | 23.04 (20.88~25.20) | 23.69 (21.47~25.91) |
| **Women** |  |  |  |  |
| Q1 | 36.17 (27.87~44.47) | 37.21 (29.10~45.32) | 43.60 (35.17~52.03) | 46.39 (37.41~55.37) |
| Q2 | 36.31 (27.59~45.03) | 37.26 (27.84~46.68) | 43.79 (34.24~53.34) | 47.50 (37.84~57.16) |
| Q3 | 35.82 (27.09~44.55) | 37.63 (27.12~48.14) | 41.73 (30.48~52.98) | 46.81 (36.47~57.15) |
| Q4 | 36.34 (24.92~47.76) | 36.54 (24.87~48.21) | 40.86 (27.92~53.80) | 47.04 (35.59~58.49) |

**Table A4.** The regression based adjusted smoking intensity by SES quartile and gender.

|  | **Adjusted prevalence of smoking intensity and 95% confidence intervals (cigs/day)** | | | |
| --- | --- | --- | --- | --- |
|  | **2012** | **2014** | **2016** | **2018** |
| **Men** |  |  |  |  |
| Q1 | 17.22 (16.74~17.70) | 17.42 (16.92~17.92) | 17.14 (16.63~17.65) | 17.04 (16.53~17.55) |
| Q2 | 16.92 (16.44~17.40) | 16.35 (15.84~16.86) | 16.22 (15.72~16.72) | 15.91 (15.41~16.41) |
| Q3 | 16.55 (16.07~17.03) | 15.68 (15.20~16.16) | 15.27 (14.74~15.80) | 14.63 (14.11~15.15) |
| Q4 | 15.88 (15.40~16.36) | 14.85 (14.33~15.37) | 14.70 (14.19~15.21) | 13.54 (13.03~14.05) |
| **Women** |  |  |  |  |
| Q1 | 13.99 (11.97~16.01) | 13.53 (11.74~15.32) | 13.08 (11.53~14.63) | 13.02 (10.48~15.56) |
| Q2 | 13.36 (11.09~15.63) | 12.41 (10.70~14.12) | 12.00 (9.95~14.05) | 12.01 (9.71~14.31) |
| Q3 | 12.77 (10.54~15.00) | 11.52 (8.88~14.16) | 10.28 (8.37~12.19) | 10.00 (8.06~11.94) |
| Q4 | 12.35 (9.84~14.86) | 10.45 (7.00~13.90) | 9.29 (6.17~12.41) | 8.13 (5.76~10.50) |

**Table A5.** The crude prevalence of current smoking by SES quartile and gender.

|  | **Prevalence of current smoking and 95% confidence intervals (%)** | | | |
| --- | --- | --- | --- | --- |
|  | **2012** | **2014** | **2016** | **2018** |
| **Men** |  |  |  |  |
| Q1 | 64.74 (62.93~66.57) | 63.29 (61.41~65.17) | 61.84 (59.96~63.73) | 61.73 (59.77~63.70) |
| Q2 | 63.95 (62.12~65.78) | 60.87 (58.96~62.78) | 59.11 (57.18~61.04) | 61.37 (59.38~63.35) |
| Q3 | 59.58 (57.71~61.44) | 58.01 (56.08~59.95) | 57.75 (55.81~59.70) | 61.47 (59.47~63.48) |
| Q4 | 56.88 (54.99~58.76) | 49.16 (47.18~51.14) | 49.13(47.13~51.14) | 48.75 (46.66~50.85) |
| **Women** |  |  |  |  |
| Q1 | 3.06 (2.43~3.70) | 3.67 (2.99~4.43) | 3.80 (3.05~4.54) | 3.46 (2.73~4.19) |
| Q2 | 3.13 (2.48~3.77) | 2.17 (1.61~2.73) | 2.31 (1.82~2.91) | 3.03 (2.32~3.74) |
| Q3 | 2.29 (1.73~2.84) | 1.66 (1.16~2.16) | 1.95 (1.39~2.51) | 2.19 (1.57~2.81) |
| Q4 | 1.00 (0.62~1.35) | 1.89 (1.36~2.42) | 1.63 (1.11~2.16) | 1.68 (1.12~2.23) |

**Table A6.** The crude prevalence of 2-year smoking cessation rates by SES quartile and gender.

|  | **Prevalence of cessation rates and 95% confidence intervals (%)** | | | |
| --- | --- | --- | --- | --- |
|  | **2012** | **2014** | **2016** | **2018** |
| **Men** |  |  |  |  |
| Q1 | 16.90 (15.28~18.52) | 22.04 (20.25~23.84) | 24.05 (22.20~25.89) | 24.51 (22.60~26.43) |
| Q2 | 17.06 (15.44~18.68) | 20.91 (19.10~22.73) | 26.13 (24.19~28.07) | 23.94 (22.01~25.87) |
| Q3 | 19.17 (17.45~20.89) | 20.79 (18.97~22.62) | 22.85 (20.94~24.76) | 22.78 (20.85~24.72) |
| Q4 | 17.07 (15.38~18.77) | 22.71 (20.69~24.73) | 24.80 (22.65~26.96) | 25.28 (23.05~27.50) |
| **Women** |  |  |  |  |
| Q1 | 43.50 (35.12~51.87) | 34.55 (26.36~42.74) | 38.59 (30.08~47.10) | 48.64 (39.56~57.71) |
| Q2 | 28.56 (19.73~37.38) | 44.81 (35.28~54.34) | 47.84 (38.17~57.50) | 37.99 (28.21~47.77) |
| Q3 | 48.76 (39.95~57.57) | 40.86 (30.19~51.52) | 40.34 (28.90~51.79) | 54.67 (44.19~65.16) |
| Q4 | 29.30 (17.65~40.95) | 42.75 (30.87~54.63) | 42.30 (29.07~55.52) | 53.00 (41.36~64.64) |

**Table A7.** The crude Smoking intensity by SES quartile and gender.

|  | **Prevalence of cessation rates and 95% confidence intervals (cigs/day)** | | | |
| --- | --- | --- | --- | --- |
|  | **2012** | **2014** | **2016** | **2018** |
| **Men** |  |  |  |  |
| Q1 | 17.13 (16.64~17.61) | 17.84 (17.33~18.34) | 17.25 (16.75~17.76) | 17.00 (16.49~17.50) |
| Q2 | 16.77 (16.29~17.25) | 16.76 (16.25~17.26) | 16.79 (16.29~17.28) | 16.50 (16.01~17.00) |
| Q3 | 17.18 (16.69~17.67) | 15.62 (15.14~16.09) | 15.60 (15.07~16.12) | 14.90 (14.38~15.42) |
| Q4 | 15.77 (15.29~16.25) | 14.87 (14.35~15.39) | 14.47 (13.95~14.98) | 13.67 (13.15~14.18) |
| **Women** |  |  |  |  |
| Q1 | 12.86 (10.81~14.91) | 12.49 (10.68~14.30) | 11.95 (10.37~13.53) | 13.26 (10.67~15.84) |
| Q2 | 12.71 (10.39~15.02) | 10.09 (8.35~11.83) | 13.06 (10.97~15.14) | 11.40 (9.05~13.75) |
| Q3 | 15.48 (13.21~17.75) | 13.84 (11.13~16.54) | 8.63 (6.67~10.60) | 9.48 (7.48~11.47) |
| Q4 | 12.44 (9.86~15.02) | 13.02 (9.46~16.58) | 9.85 (6.60~13.10) | 8.57 (6.10~11.03) |

**Annualized Cessation Rate**

The cessation rate was estimated by dividing the number of quitters at the end of the follow-up by the number of current smokers before the follow-up. Therefore, the cessation rate in this study was the self-reported point cessation prevalence. To facilitate comparisons with different studies, we estimated the annualized cessation rate ($q$) based on an assumption of a constant quit rate during the follow-up period:

$q=1-exp(\frac{\ln\left( 1-\frac{nq}{ns} \right)}{yr})$ (Eq. 1)

in which $ns$ refers to the number of smokers before the follow-up, $nq$ refers to the number of quitters at the end of the follow-up, and $yr$ refers to the number of follow-up years. The annualized cessation rate has been used in previous studies with different years of follow-up[3].

The defined smoking cessation rate in the Global Adult Tobacco Survey of China in 2018 was 15.6% (men: 15.3%; women: 23.0%), which was surprisingly similar to our findings[4].

**Table A8.** The 2-year and annualized cessation rates by gender.

| **Gender** | **Year** | **2-year cessation rate**  **(%)** | **Annualized cessation rate**  **(%)** |
| --- | --- | --- | --- |
| **Men** | 2012 | 17.55 | 9.20 |
|  | 2014 | 21.61 | 11.46 |
|  | 2016 | 24.45 | 13.08 |
|  | 2018 | 24.08 | 12.87 |
| **Women** | 2012 | 39.44 | 22.18 |
|  | 2014 | 40.28 | 22.72 |
|  | 2016 | 42.08 | 23.89 |
|  | 2018 | 48.39 | 28.16 |
| **Total** | 2012 | 18.68 | 9.82 |
|  | 2014 | 22.57 | 12.01 |
|  | 2016 | 25.39 | 13.62 |
|  | 2018 | 25.47 | 13.67 |

1. Vyas S, Kumaranayake L: Constructing socio-economic status indices: how to use principal components analysis. *Health Policy Plan* 2006, 21(6):459-468.

2. Li C: Prestige Stratification in the Contemporary China:Occupational prestige measures and socio-economic index. *Sociological Research* 2005, 2:74-102.

3. Hyland A, Li Q, Bauer JE, Giovino GA, Steger C, Cummings KM: Predictors of cessation in a cohort of current and former smokers followed over 13 years. *Nicotine Tob Res* 2004, 6 Suppl 3:S363-369.

4. CDC: Report of 2018 Adult Smoking Survey in China. Beijing: China Centers for Disease Control and Prevention; 2019.
